# Supplementary material for: Chronic and immediate refined carbohydrate consumption and facial attractiveness
Source: PLoS One. 2024 Mar 6;19(3):e0298984. doi: 10.1371/journal.pone.0298984 (PMC10917283; doi:10.1371/journal.pone.0298984)
Supplement: S1 Table — (DOCX) [file pone.0298984.s001.docx]

**Table S1**. Exhaustive list of the different food items of the diet questionnaire in French and translated.

| French | English |
| --- | --- |
| Baguette (morceaux)  Pain (morceaux)  Pain de campagne  Pain complet  Pain de mie  Pain de seigle  Pain poilane  Pain découpé (restaurant-cantine)  Couleur café au lait  Couleur chocolat au lait  Blé soufflé  Müesli  Pétales de maïs  Biscottes diverses  Brioches tranches ou individuelles  Beurre  Epaisseur du beurre  Confiture (consommation totale, e.g. tartine, boisson, laitage)  Miel (consommation totale y compris tartine, boisson, laitage)  Nutella (consommation totale y compris tartine, boisson, laitage)  Sucre morceaux (consommation totale y compris boisson, laitage)  Sucre poudre (consommation totale y compris boisson, laitage)  Chèvre crottin  Bûche  Pyramide  Boursin  Camembert  Rouy  Gruyère  Mimolette  Roquefort  Tomme  Fromage blanc  Nombre de yaourts déjà sucrés à l'achat (aromatisé, aux fruits)  Nombre de yaourts natures  Nombre de petits-suisses nature  Yaourt à boire: nombre de verres  Taille du verre pour le yaourt à boire  Chantilly  Entremet semoule et riz  Flan  Mousse au chocolat  Gâteau à la crème  Gâteau mousse aux fruits  Tarte aux fruits  Gâteau au chocolat  Tourte aux amandes  Cake  Madeleines  Quatre-quarts  Type de biscuits consommés  Nombre de biscuits consommés  Nombre de viennoiseries consommées (croissant, pain au chocolat,…)  Noix  Noisettes  Pistaches  Cerises  Fraises  Framboises  Banane  Pomme  Poire  Kiwi  Abricot  Groseilles  Quetsches (prunes)  Raisins  Orange  Pruneaux  Ananas au sirop  Fruis au sirops divers  Bonbons divers  Chocolat (carrés)  Barre chocolatée  Soda, jus de fruit: nombre de boissons individuelles consommées (canette-bouteille-brick)  Type de contenant de boisson individuelle (canette-bouteille-brick)  Soda, jus de fruit: nombre de verres  Taille du verre (soda ou jus)  Taille de la tasse ou du bol (lait-café-chocolat) | Baguette (pieces)  Bread (pieces)  Country bread  Whole wheat bread  Soft bread  Rye bread  Poilane bread  Sliced ​​bread (restaurant-canteen)  Latte coffee color  Milk chocolate color  Puffed wheat  Muesli  Corn petals  Various rusks  Sliced ​​or individual brioches  Butter  Butter thickness  Jam (total consumption including for instance toast, drink, dairy)  Honey (total consumption including toast, drink, dairy)  Nutella (total consumption including toast, drink, dairy)  Sugar cubes (total consumption including drink, dairy)  Granulated sugar (total consumption including drink, dairy)  Goat cheese  Goat cheese  Goat cheese  Boursin  Camembert  Rouy  Gruyere  Mimolette  Roquefort  Cheese  White cheese  Number of yogurts already sweetened (flavored, with fruits)  Number of plain yogurts  Number of plain Petits-Suisses  Drinkable yogurt: number of glasses  Glass size for yogurt drink  Sweet whipped cream  Semolina and rice dessert  Blank  Chocolate mousse  Cream cake  Fruit mousse cake  Fruit tart  Chocolate cake  Almond pie  Cake  Madeleines  Pound cake  Type of cookies eaten  Number of cookies eaten  Number of pastries consumed (croissant, pain au chocolat, etc.)  Nut  Hazelnut  Pistachios  Cherries  Strawberries  Raspberries  Banana  Apple  Pear  Kiwi  Apricot  Currants  Quetsches (plums)  Grapes  Orange  Prunes  Pineapple in syrup  Fruits in various syrups  Various candies  Chocolate (squares)  Chocolate bar  Soda, fruit juice: number of individual drinks consumed (can-bottle-brick)  Type of individual beverage container (can-bottle-brick)  Soda, fruit juice: number of glasses  Glass size (soda or juice)  Cup or bowl size (milk-coffee-chocolate) |
