# Supplementary material for: Chronic and immediate refined carbohydrate consumption and facial attractiveness
Source: PLoS One. 2024 Mar 6;19(3):e0298984. doi: 10.1371/journal.pone.0298984 (PMC10917283; doi:10.1371/journal.pone.0298984)
Supplement: S2 Table — Raters were instructed to ascribe an age for the photographs they were viewing. The estimate (β), standard error of the mean (se), χ² statistic, and corresponding p-value are given. Bold characters indicate significant (p < 0.05) effects. (DOCX) [file pone.0298984.s002.docx]

**Table S2**. Effects of rater characteristics and subject age and sex on the subject age perception by raters. Raters were instructed to ascribe an age for the photographs they were viewing. The estimate (β), standard error of the mean (se), χ² statistic, and corresponding p-value are given. Bold characters indicate significant (p < 0.05) effects.

|  | Male faces (N = 52) | | | |  | Female faces (N = 52) | | | |
| --- | --- | --- | --- | --- | --- | --- | --- | --- | --- |
|  | β | se | χ² | p |  | β | se | χ² | p |
| Intercept | 25.5 | 0.41 |  |  |  | 24.6 | 0.38 |  |  |
| Subject age | 1.36 | 0.28 | 22.6 | **10^-6^** |  | 0.95 | 0.24 | 15.8 | **10^-5^** |
| Rater age | - 0.26 | 0.26 | 0.95 | 0.32 |  | - 0.36 | 0.24 | 2.26 | 0.13 |
| Rater sex | - 0.04 | 0.45 | 0.01 | 0.93 |  | 0.21 | 0.44 | 0.23 | 0.62 |
| Rater study level | - 0.25 | 0.26 | 0.94 | 0.33 |  | 0.21 | 0.25 | 0.71 | 0.39 |
