# Supplementary material for: Chronic and immediate refined carbohydrate consumption and facial attractiveness
Source: PLoS One. 2024 Mar 6;19(3):e0298984. doi: 10.1371/journal.pone.0298984 (PMC10917283; doi:10.1371/journal.pone.0298984)
Supplement: S3 Table — The Wilcoxon test statistic (V), Friedman chi-squared (F) and corresponding p-value are given. Bold characters indicate significant (p < 0.05) effects. Median and terciles of age and study level were used for the Wilcoxon signed-rank test and Friedman two-way analysis of variance, respectively. (DOCX) [file pone.0298984.s003.docx]

**Table S3.** Effects of rater characteristics on the subjects’ masculinity/femininity perception by raters. The Wilcoxon test statistic (V), Friedman chi-squared (F) and corresponding p-value are given. Bold characters indicate significant (p < 0.05) effects. Median and terciles of age and study level were used for the Wilcoxon signed-rank test and Friedman two-way analysis of variance, respectively.

|  |  | Men faces (N = 52) | | |  | Women faces (N = 52) | |
| --- | --- | --- | --- | --- | --- | --- | --- |
|  |  | | V | p-value |  | V | p-value |
| Wilcoxon signed-rank test | Rater age | | 593 | 0.963 |  | 582 | 0.332 |
|  | Rater study level | | 671 | 0.944 |  | 507 | 0.294 |
|  |  | | F(χ²) | p-value |  | F(χ²) | p-value |
| Friedman two-way analysis of variance | Rater age | | 4.657 | 0.097 |  | 2.261 | 0.323 |
|  | Rater study level | | 1.107 | 0.575 |  | 0.131 | 0.937 |
