# Supplementary material for: Chronic and immediate refined carbohydrate consumption and facial attractiveness
Source: PLoS One. 2024 Mar 6;19(3):e0298984. doi: 10.1371/journal.pone.0298984 (PMC10917283; doi:10.1371/journal.pone.0298984)
Supplement: S4 Table — The Wilcoxon test statistic (V), Friedman chi-squared (F) and corresponding p-value are given. Bold characters indicate significant (p < 0.05) effects. Median and terciles of age and study level were used for the Wilcoxon signed-rank test and Friedman two-way analysis of variance, respectively. (DOCX) [file pone.0298984.s004.docx]

**Table S4.** Effects of rater characteristics on the subjects’ attractiveness perception by raters. The Wilcoxon test statistic (V), Friedman chi-squared (F) and corresponding p- value are given. Bold characters indicate significant (p < 0.05) effects. Median and terciles of age and study level were used for the Wilcoxon signed-rank test and Friedman two-way analysis of variance, respectively.

|  |  | Men faces (N = 52) | | |  | Women faces (N = 52) | |
| --- | --- | --- | --- | --- | --- | --- | --- |
|  |  | | V | p-value |  | V | p-value |
| Wilcoxon signed-rank test | Rater age | | 588 | 0.360 |  | 650 | 0.712 |
|  | Rater study level | | 697 | 0.945 |  | 735.5 | 0.675 |
|  |  | | F(χ²) | p-value |  | F(χ²) | p-value |
| Friedman two-way analysis of variance | Rater age | | 1.459 | 0.482 |  | 0.907 | 0.635 |
|  | Rater study level | | 0.269 | 0.874 |  | 0.299 | 0.860 |
