# Supplementary material for: Chronic and immediate refined carbohydrate consumption and facial attractiveness
Source: PLoS One. 2024 Mar 6;19(3):e0298984. doi: 10.1371/journal.pone.0298984 (PMC10917283; doi:10.1371/journal.pone.0298984)
Supplement: S5 Table — The results based on Figs 2 and 3. RC1, RC2 and RC3 are the three variables representing refined carbohydrate consumption. The standardized estimate (β), standard error of the mean (se), z-value, and corresponding p-value are given. Bold characters indicate significant (p < 0.05) effects. Foxing each sex, only variables with a p-value < 0.01 were integrated into the model. (DOCX) [file pone.0298984.s005.docx]

**Table S5**. Structural equation analysis. The results based on Figures 2 and 3. RGL1, RGL2 and RGL3 are the three variables representing refined carbohydrate consumption. The standardized estimate (β), standard error of the mean (se), z-value, and corresponding p-value are given. Bold characters indicate significant (p < 0.05) effects. Foxing each sex, only variables with a p-value < 0.01 were integrated into the model.

|  | | Male faces evaluated by women | | | |  | Female faces evaluated by men | | | |
| --- | --- | --- | --- | --- | --- | --- | --- | --- | --- | --- |
| Regressions~ |  | β | se | z-value | p |  | β | se | z-value | p |
| Attractiveness | RGL1 |  |  |  |  |  | - 0.127 | 0.104 | - 1.228 | 0.220 |
|  | RGL2 | 0.077 | 0.119 | 0.644 | 0.519 |  | - 0.101 | 0.110 | - 0.921 | 0.357 |
|  | RGL3 | - 0.011 | 0.130 | - 0.088 | 0.930 |  | - 0.047 | 0.105 | - 0.452 | 0.651 |
|  | EI1 | 0.108 | 0.123 | 0.880 | 0.379 |  | 0.161 | 0.105 | 1.531 | 0.126 |
|  | EI2 | 0.064 | 0.130 | 0.491 | 0.623 |  |  |  |  |  |
|  | Breakfast type | - 0.204 | 0.122 | - 1.675 | 0.094 |  | 0.092 | 0.105 | 0.875 | 0.382 |
|  | Age | 0.081 | 0.130 | 0.618 | 0.537 |  | - 0.175 | 0.105 | -1.668 | 0.095 |
|  | Age departure |  |  |  |  |  | - 0.102 | 0.110 | -0.924 | 0.356 |
|  | Fem/Masc Index | - 0.137 | 0.121 | - 1.134 | 0.257 |  |  |  |  |  |
|  | Perceived masculinity/femininity | 0.268 | 0.139 | 1.933 | 0.053 |  | 0.414 | 0.103 | 4.021 | **< 10^-3^** |
|  | Physical activity | 0.214 | 0.134 | 1.599 | 0.110 |  | 0.277 | 0.107 | 2.597 | **0.009** |
|  | Parental home ownership |  |  |  |  |  | 0.216 | 0.105 | 2.056 | **0.040** |
|  | Contraceptive |  |  |  |  |  | - 0.186 | 0.106 | - 1.758 | 0.079 |
|  | Couple status | 0.099 | 0.131 | 0.754 | 0.451 |  |  |  |  |  |
|  | Facial hairiness | - 0.159 | 0.128 | - 1.242 | 0.214 |  |  |  |  |  |
| Age departure | RGL1 |  |  |  |  |  | - 0.081 | 0.129 | - 0.625 | 0.532 |
|  | RGL2 |  |  |  |  |  | 0.306 | 0.124 | 2.475 | **0.013** |
|  | RGL3 |  |  |  |  |  | - 0.165 | 0.128 | - 1.286 | 0.198 |
| Perceived masculinity/femininity | RGL1 |  |  |  |  |  | - 0.007 | 0.136 | - 0.052 | 0.959 |
|  | RGL2 | - 0.029 | 0.115 | - 0.249 | 0.803 |  | - 0.054 | 0.136 | - 0.396 | 0.692 |
|  | RGL3 | - 0.275 | 0.113 | - 2.441 | **0.015** |  | - 0.055 | 0.135 | - 0.408 | 0.683 |
|  | EI1 | 0.216 | 0.114 | 1.904 | 0.057 |  | 0.013 | 0.136 | 0.097 | 0.923 |
|  | EI2 | - 0.366 | 0.109 | - 3.357 | **0.001** |  |  |  |  |  |
|  | Breakfast type | - 0.130 | 0.115 | - 1.132 | 0.258 |  | - 0.195 | 0.132 | - 1.479 | 0.139 |
|  | Facial hairiness | 0.185 | 0.113 | 1.633 | 0.102 |  |  |  |  |  |
| Contraceptive | Perceived femininity |  |  |  |  |  | - 0.247 | 0.130 | - 1.896 | 0.058 |
| RGL1 | Physical activity |  |  |  |  |  | 0.063 | 0.138 | 0.458 | 0.647 |
| RGL2 | Physical activity | - 0.009 | 0.139 | - 0.065 | 0.948 |  | - 0.179 | 0.133 | - 1.343 | 0.179 |
| RGL3 | Physical activity | - 0.304 | 0.123 | - 2.470 | **0.014** |  | - 0.027 | 0.139 | - 0.195 | 0.845 |
| EI1 | Physical activity | 0.100 | 0.137 | 0.727 | 0.467 |  | 0.227 | 0.130 | 1.749 | 0.080 |
| EI2 | Physical activity | - 0.049 | 0.138 | - 0.356 | 0.722 |  |  |  |  |  |
| Fem/Masc Index | RGL2 | 0.157 | 0.133 | 1.179 | 0.238 |  |  |  |  |  |
|  | RGL3 | - 0.061 | 0.135 | - 0.453 | 0.651 |  |  |  |  |  |
|  | EI1 | - 0.107 | 0.134 | - 0.798 | 0.425 |  |  |  |  |  |
|  | EI2 | 0.122 | 0.134 | 0.908 | 0.364 |  |  |  |  |  |
