# Supplementary material for: Chronic and immediate refined carbohydrate consumption and facial attractiveness
Source: PLoS One. 2024 Mar 6;19(3):e0298984. doi: 10.1371/journal.pone.0298984 (PMC10917283; doi:10.1371/journal.pone.0298984)
Supplement: S6 Table — After a random 10% data reduction, p-values are computed and this process is repeated 1000 times, providing a p-value distribution for each variable. RGL1, RGL2 and RGL3 are the three variables representing refined carbohydrate consumption. The mean p-value (mean p), standard deviation (sd), minimum p-value (min) and maximum p-value (max) are given. (DOCX) [file pone.0298984.s006.docx]

**Table S6**. Sensitivity analysis for the test of attractiveness for male or female faces. After a random 10% data reduction, p-values are computed and this process is repeated 1000 times, providing a p-value distribution for each variable. RGL1, RGL2 and RGL3 are the three variables representing refined carbohydrate consumption. The mean p-value (mean p), standard deviation (sd), minimum p-value (min) and maximum p-value (max) are given.

|  | Male faces evaluated by women | | | |  | Female faces evaluated by men | | | |
| --- | --- | --- | --- | --- | --- | --- | --- | --- | --- |
|  | mean p | sd | min | max |  | mean p | sd | min | max |
| RGL1 | 0.789 | 0.151 | 0.276 | 0.999 |  | 0.037 | 0.037 | 6 10^-4^ | 0.363 |
| RGL2 | 6 10^-6^ | 10^-5^ | 2 10^-8^ | 10^-4^ |  | 0.058 | 0.047 | 0.002 | 0.460 |
| RGL3 | 10^-6^ | 4 10^-6^ | 2 10^-11^ | 5 10^-5^ |  | 0.139 | 0.097 | 0.004 | 0.547 |
| EI1 | 3 10^-5^ | 8 10^-5^ | 2 10^-8^ | 0.001 |  | 3 10^-4^ | 4 10^-4^ | 8 10^-6^ | 0.006 |
| EI2 | 6 10^-4^ | 8 10^-4^ | 3 10^-6^ | 0.010 |  | 0.783 | 0.152 | 0.270 | 0.999 |
| EI3 | 0.688 | 0.179 | 0.187 | 0.999 |  | 0.394 | 0.166 | 0.057 | 0.999 |
| Breakfast type | 10^-5^ | 2 10^-5^ | 4 10^-9^ | 2 10^-4^ |  | 10^-8^ | 8 10^-8^ | 2 10^-12^ | 2 10^-6^ |
| Age | 0.001 | 2 10^-3^ | 5 10^-6^ | 0.039 |  | 5 10^-4^ | 0.001 | 4 10^-7^ | 0.014 |
| Age departure from actual age | 0.212 | 0.152 | 0.011 | 0.970 |  | 2 10^-4^ | 6 10^-4^ | 3 10^-8^ | 0.008 |
| Fem/Masc Index | 0.139 | 0.106 | 0.003 | 0.719 |  | 0.731 | 0.186 | 0.162 | 0.999 |
| Perceived masculinity/femininity | 0.095 | 0.060 | 0.011 | 0.492 |  | 0.007 | 0.007 | 2 10^-4^ | 0.056 |
| BMI | 0.604 | 0.197 | 0.144 | 0.999 |  | 0.638 | 0.208 | 0.107 | 0.999 |
| Physical activity | 0.095 | 0.070 | 0.004 | 0.438 |  | 0.002 | 0.003 | 4 10^-5^ | 0.029 |
| Smoker | 0.643 | 0.199 | 0.107 | 0.999 |  | 0.640 | 0.197 | 0.123 | 0.998 |
| Parental home ownership | 0.455 | 0.206 | 0.055 | 0.999 |  | 0.027 | 0.027 | 2 10^-4^ | 0.195 |
| Contraceptive | - | - | - | - |  | 3 10^-5^ | 7 10^-5^ | 6 10^-9^ | 0.001 |
| Couple status | 0.008 | 0.011 | 5 10^-5^ | 0.137 |  | 0.289 | 0.146 | 0.023 | 0.812 |
| Facial hairiness | 10^-4^ | 2 10^-4^ | 4 10^-7^ | 0.002 |  | - | - | - | - |
