# Supplementary material for: Chronic and immediate refined carbohydrate consumption and facial attractiveness
Source: PLoS One. 2024 Mar 6;19(3):e0298984. doi: 10.1371/journal.pone.0298984 (PMC10917283; doi:10.1371/journal.pone.0298984)
Supplement: Attachment — Submitted filename: response_to_reviewers.docx [file pone.0298984.s007.docx]

Thank you for the comments. They have all been taken into consideration, and the revised manuscript is significantly improved.

**Reviewer #1**: I read the whole manuscript entitled " Chronic and immediate refined carbohydrate consumption measured by glycemic load, and facial attractiveness" and I have few suggestions.

- It's very hard to state attractiveness in words but it would be great if authors can define in few words/lines about it. Also, the opposite genders always have some degree of attractiveness to each other too. So, a formal statement about it should also be included in definition.

A sentence was added page 3, lines 76-78: “In the field of evolutionary biology, attractiveness (or preference) refers to an individual's tendency to be drawn to specific traits or characteristics in potential mating or social exchange partners.”

- Page#3 and Line#64: I suggest changing the word “repeated” with persistent.

This word has been changed as suggested.

- Page#3 and Line#68-69: any reference to support this would be great

We have added a reference page 3, line 67.

**Reviewer #2:** Thank you for the opportunity to review this very interesting study.
This is an observational study assessing the association between refined carbohydrate consumption and facial attractiveness. The authors have conducted a thorough investigation, taking into account a large number of variables that could potentially be related to the main outcome. Although I find great interest in the study and its results, I have some concerns regarding the methodology and study design. I find that the authors have not controlled well all possible confounders and have conducted a large number of tests in order to explore various associations. Also, the sample population is very small in order to make reliable conclusions about the primary outcome if all possible confounders are taken into account. Therefore, I am unfortunately not able to recommend publication of this manuscript in its current form. My detailed comments are listed below.

Major comments:

**•Introduction**

- I suggest that the authors rephrase and soften their statements about the association between carbohydrate consumption and medical disease. Despite current evidence regarding these relationships, most of these diseases have multifaceted etiologies and are likely not attributed to a single cause. I am referring to the first paragraph of the introduction.

In the first paragraph of introduction, we have moderated the importance of the link between refined carbohydrate consumption and medical problems page 3, lines 58-62: “The mismatch between how human physiology has evolved and Western industrialized lifestyles is seen as a contributing factor to the current epidemic of numerous medical problems. For example, it has been shown that this massive dietary change was involved in obesity, insulin resistance, type II diabetes, cardiovascular diseases, Alzheimer’s disease, hypertension or myopia (2–5).”

- The authors are citing Reference #8 to support a lot of their statement in the introduction. Reference #8 is a non-systematic, topic review article with little scientific merit. I suggest that the authors support their statements with research articles or systematic reviews/meta-analyses throughout the manuscript.

We have added reference to research articles or reviews throughout the manuscript each time this reference is cited (it is now reference #12). We agree that this reference is not a systematic review or meta-analyses. However, Cordain et al 2003 were the first to propose a plausible synthesis about the potential hormonal consequences of refined carbohydrate consumption on health.

**•Material and Methods**

- Please provide more information about the study population in the text. There is no information regarding the number of subjects per group, ages, sexual orientation, etc. This information are only partially provided in tables. It appears that references #27 is a study previously performed by the group using the same sample population. I looked at the study, but the demographic information provided there is also incomplete. Please elaborate in detail on the characteristics of the study population.

Characteristics of the population are now provided in the Materials and Methods section, page 6, lines 138-144, and in the new Table 1 (corresponding to the former Table 4, completed). The order of the tables has been changed and the first paragraph of the Results section has been deleted. The population information contained in this paragraph has been moved to the Materials and Methods section for the sake of consistency (lines 165-167, page 8; lines 210-213,page 10; lines 234-235, page 11).

- In study #27, the authors mention that the study population comprises young adults between 20-30 yrs of age. This information is not provided clearly in the present manuscript, however I assume that is true. There is a large discrepancy in the age of the subjects and the raters. It is well documented that age has a significant effect on the perception of attractiveness, with older individuals becoming “less strict” with time.

This information on age of the study population in now clearly provided in Materials and Methods section, line 138 page 6. Effect of rater’s age on attractiveness (and also level study) was tested and was non significant for both sex. This is now indicated in manuscript page 13, lines 285-297, in a new paragraph “*Effects of rater characteristics on their attractiveness perception”* and results have been added in a new table in Supporting Information, Table S4.

- The authors report keeping the first 16 PDs for their shape analysis (explaining more than 83% of variation). How did they decide on this cut off limit? Was the broken-stick method used?

The procedure is now explained in the text page 9 line 189-190: “ An arbitrary cut-off of minimum 80% variance explained was applied to select the axis, thus the first 16 axes were retained (explaining 83.7% of variance) for further analyses”.

- There is no mention in the manuscript regarding the assessment of systematic and random error related to the study methodology. In my view this is an essential part of the study methodology when conducting studies that investigate the subjective outcomes such as facial attractiveness.

We understand the importance of assessing systematic and random errors in studies investigating subjective outcomes such as facial attractiveness. While these aspects were not explicitly addressed in the first version of the manuscript, we would like to highlight that we have taken measures to account for them in our methodology. To assess systematic errors, we meticulously designed our study by following established protocols and ensuring that experimental conditions were standardized as much as possible (for subjects and raters). For example, we used recruitment procedures to limit the risk of selection bias by defining clear inclusion and exclusion criteria for our study samples. In addition, all photographs and all assessments of facial attractiveness were conducted in a controlled environment with consistent lighting, background, and room temperature to reduce variability caused by external factors. Participants and raters were also kept blind to the study's objectives to minimize potential biases related to knowledge of the research goals. Additionally, we employed appropriate statistical methods to control for potential confounding factors (for raters and subjects). Regarding random errors, we used reliable and reproducible measurement techniques for the photographs and the assessment of facial attractiveness. The sample sizes of participants and raters were determined on the basis of power calculations to ensure that the study had sufficient statistical power to detect significant differences in attractiveness ratings and we increased the sample sizes to minimize the impact of random variability (subjects and raters). This is now included in the manuscript in materials and methods, section “*Assessment of systematic and random errors.*“ (lines 339-356 page 15).

**•Results**

- It appears that a 10% type-1 error was accepted for all statistical analyses. Can the authors please elaborate on this decision?
Type-I error is 5% throughout. There were several mentions in the text of “marginally significant” results when the P-value was between 0.05 and 0.10. To avoid confusion, these situations are now mentioned as “marginally non-significant” (page 16, lines 377, 389; page 17, lines 392, 405, 411). See also below for the path-analysis section (Minor comments / Figures and Tables).

Minor comments:

**•Title**

-The title is a little confusing. It implies that carbohydrate consumption was measured with facial attractiveness. I suggest rephrasing the title to: “Refined carbohydrate consumption and facial attractiveness.”

The title was rephrased: “Chronic and immediate Refined carbohydrate consumption and facial attractiveness”

**•Abstract**

- Please include more specific results in the abstract. For example, how much was facial attractiveness decreased?
More specific results are now included in the abstract, lines 38-45.

**•Ethic Statement**

- The authors state that no authors had access to identifying information of the study participants. Were the authors not part of the study protocol that was submitted to the ethics committee? Did none of the authors participate in data collection and analyses? Could the authors please provide an “author contribution statement” in the revised manuscript?

Some authors did participate at all stages of the study. Data were analyzed anonymously and no authors had access to information that could identify individual participants during or after data collection. We gave each participant a unique ID and each facial sample was anonymized with a separate set of ID. The translation key between these two sets of IDs was stored in a separate file. This file was stored in a secured server and was not stored beside the collected data.

We have had a section “Author contribution in the revised manuscript” page 25 line 591-594. “CB and MR planned and supervised study. AV, CB, LG and VD conducted different parts of study. AV and CB analyzed study. AV, CB and MR wrote the manuscript. All authors read and approved the final manuscript.”

**•Introduction:**

- There is a large number of references used in the first paragraph (2-14) to show an association between carbohydrate consumption and medical disease. I would suggest to reduce the number of references to the most relevant ones that apply more to the topic of interest.

This was done. We have reduced the number of references.

**•Material and Methods**

- Menstrual cycle has an effect on facial attractiveness in women. Facial attractiveness increases during ovulation, as rated by male observers. This is a confounding factor that was not taken into consideration. Please see: “Roberts, S. C. et al. Female facial attractiveness increases during the fertile phase of the menstrual cycle. Proceedings of the Royal Society of London. Series B: Biological Sciences 271, 1–3 (2004).’
Also, women’s perception of male facial attractiveness is influenced by their menstrual cycle which would also affect ratings in this study. Please see: Penton-Voak, I. S. et al. Menstrual cycle alters face preference. Nature 399, 741–742 (1999).
These factors need to be discussed and considered as serious confounders in the methodology of this investigation.
Facial attractiveness is indeed influenced by many variables and we have taken into account the main ones, such as age, facial morphology, hormonal contraception, etc. But of course there are many variables not considered in this study, including menstrual cycles. This is compensated by the sample size, with the hypothesis that the sample is randomized according to the variables not included. In other words, if the stage of the menstrual cycle of each women participant is randomized for the sampling day, then there is no bias of not considering this variable. This limitation is now stated in the manuscript page 24, lines 567-573 and hormonal contraception was added in the new Table 1.

- Was raters’ sexual orientation taken into consideration in the regression models or in rater selection?

In order to reduce cultural sample heterogeneity, we kept for analyses heterosexual individuals (participants and raters).

This is now clearly indicated: line 138, page 6 for participants; line 229, page 11 for masculinity/femininity raters; line 250, page 12 for attractiveness raters.

**•Figures and Tables**

- Figures 2 and 3: Is the P-value a typo or did the authors accept a 10% type-1 error?

Structural equation modeling was performed using (conservatively) the variables from the model studying perceived attractiveness displaying *P* < 0.1, as explained in the text (page 14, lines 322-323). But all results described in this study are based on type-I error of 5%. This is now clearly stated. The legends of Figures 2 and 3 have been modified to avoid confusion.

- I suggest that the authors reduce the text in the result section and add tables and figures displaying their results more visually. This will help readership the understand and interpret the results easier.

We have added a new figure (Figure 4) which is a forest plot with the odds ratio from the model studying perceived attractiveness.
