# Supplementary material for: Chronic and immediate refined carbohydrate consumption and facial attractiveness
Source: PLoS One. 2024 Mar 6;19(3):e0298984. doi: 10.1371/journal.pone.0298984 (PMC10917283; doi:10.1371/journal.pone.0298984)
Supplement: Attachment — Submitted filename: Response_to_Reviewers_2.docx [file pone.0298984.s008.docx]

Thank you for the comments. They have all been taken into consideration, and the revised manuscript is significantly improved.

**Reviewer #3:** I have carefully read the manuscript “Chronic and immediate refined carbohydrate consumption and facial attractiveness.” Note that I became a reviewer at this round; and my knowledge of the evolution of the manuscript is limited to what is present in the “response to reviewers” section. I gather that the revisions sufficiently addressed the previous concerns. I found much to like in the manuscript and found the topic novel and interesting. However, I think some further revisions may be necessary. I list potential revisions below for the editor and authors’consideration.

1. My biggest issue with the present article was that I was unable to find any report of power analyses and could not discern how sample size was determined. In the revision, the authors explain in response to a reviewer that “The sample sizes of participants and raters were determined on the basis of power calculations to ensure that the study had sufficient statistical power to detect significant differences in attractiveness ratings, based on a previous study (8) and were chosen to minimize the impact of random variability.” The same text is also in the manuscript now. I was unable to find any details of power analyses in reference #8, (which, to be sure, should be here: https://journals.sagepub.com/doi/full/10.1177/1474704920960440). If power analyses indeed exist, they need to be reported. If they do not exist, then sensitivity analyses should be provided retrospectively and their result would critically determine the evaluation of the article (my decision recommendation of "minor revision" would probably not hold any longer). The current sample size is not so large by some standards (e.g., https://www.sciencedirect.com/science/article/pii/S0092656613000858) and we need to have a clearer idea of the reliability of the present findings.

The explanation of the choice of sample size based on our pilot study has been given in lines 350 to 352, page 15, and the sentence has been reworded. We agree that our sample size is not very large. This is why we now present the details of the sensibility analysis (randomly deleting 10% of the data and computing p-values, then repeating this process 1000 times, and analyzing the resulting p-value distributions for each variable) in the new Table S6.

2. My position amidst the incessant debates about null hypothesis significance testing is that usage of phrases such as “marginally significant” are in error. If you subscribe to the Neyman-Pearson approach, there is no such thing. I would recommend removing this phrase and being consistent in the usage of alpha level to decide on significance and its absence. In addition, there are many p-values derived from the same dataset and no consideration of experiment wise Type I error. Thus, if one also extends the threshold for deciding on the presence of effects to the “marginal” area (e.g., .05-.1), then there is an even greater risk of some of these findings to represent Type 1 error.

We agree. The only occurrence of “marginally significant” was replaced with “marginally non-significant” (line 501 page 22). Thus, the threshold of significance is maintained at 0.05 throughout. Thus type-I error are not inflated due to a variable threshold.

3. I did not see any mention of sleep quality (chronic and in the night prior to data collection) but based on the literature, this is another important factor in the current setting and could be mentioned as something that future studies should aim to control.

We now mention this point in the limitations section and have added a reference.

4. I did not see any mention of make-up or facial accessories. Was there any request for participants to remove make-up or detachable accessories (piercing, etc.)? If not this would be a serious limitation and could also be confounded with lifestyle and thus the chronic diet. Did any participants have facial tattoos? At the least, I would expect the authors to handle this by performing the same kind of procedure they applied to facial hairiness.

We indeed asked participant to remove their make-up and all detachable accessories. In the manuscript, we already stated on page 6, line 135 to 136 “The subjects were asked to express a neutral face (without a smile), to tie their hair and to remove any glasses or earrings”. We added clarifications as suggested “The subjects were asked to express a neutral face (without a smile), to tie their hair and to remove any glasses, earrings, piercing or make-up.” No participant was wearing a facial tattoo. Indeed, absence of facial tattoo was a condition for participation, and this is now stated (line 115, page 5).

5. Response options for the “geographical origin of the grandparents” item could be added. It is not clear whether this is asking for ethnicity directly or the researchers are inferring ethnicity from geographical position of where the grandparents were born or grew up in.

We asked participants to specify the continent each of their grandparents came from in order to reduce cultural heterogeneity (only participants with their 4 grandparents of European origin were selected). The text has been modified to specify this aspect (line 120-121 page 5).

6. Line 191: The full term for “LDA” should be added (i.e., “linear discriminant analysis” I suppose).

This was done. We added the full term.

7. The authors describe where participants were approached but not where they were tested. The latter should be added.

We added this page 5 lines 116 and replaced the sentence “Subjects were given an appointment early in the morning and had to come in on an empty stomach in groups of three to four” with “Subjects were given an early-morning appointment and were asked to come to our laboratory for the experiments in groups of three or four on an empty stomach”.

8. Where only p-values are provided, full statistics could be added, such as on lines 234-235 (correlation coefficients should be added there). Assuming those are p-values, why are they capitalized?

We have replaced “P” with “p” for p-values throughout the manuscript. We have added the full statistics when p-values were provided without a table.

9. I think the Statistical Analyses section would be easier to read if there was an overview of what was done and why at the beginning (and/or at the beginning of each of its subsections). Why was a particular analysis needed could be made clearer. In addition, I think the subsections should be grouped under broader headings. Which subsections are preparatory or necessary checks and which ones contain the central tests would become clearer this way.

An overview of what was done and why has been added lines 261 to 264 page 12. A more general heading has also been added above the subsections devoting to raters’ characteristics.

10. It is not clear to me why parental home ownership was assessed. I assume it is a proxy for the participant’s socioeconomic status as these are relatively young individuals. Whatever it is assumed to measure, how is it linked to facial attractiveness (or how is it relevant to the current study)? These could be clarified.

Indeed, parental home ownership was assessed as a proxy of the socioeconomic status of the young participants. Higher socioeconomic status may provide individuals with better access to resources, including healthcare, nutrition or education. This can contribute to overall health and well-being, potentially influencing facial features associated with attractiveness. Moreover, individuals may be attracted to traits associated with higher socioeconomic status due to perceptions of stability, resources and overall fitness. Socioeconomic status is therefore potentially linked to attractiveness and diet. This is now precised line 122 page 5 and a reference has been added page 14 line 313.

11. In the parenthesis that starts at the end of line 310, shouldn’t one of the three modalities be “B2 versus B1” (instead of two of them both being “B1 versus B2”)?

Thank you, it was a mistake. This is now corrected.

12. In the captions for Figures 2 and 3, instead of “women/men to explain their attractiveness,” one could simply write “women’s/men’s attractiveness.”

This has been modified.

13. Line 398: Shouldn’t “total deviance” be “total variance?”

As we have used logistic regression, we must thus speak of deviance rather than variance.

14. Line 462: “influence” should be “influences”. In the same line, as in others, I think it’s better to follow an earlier reviewer’s suggestion of using “persistent” or “chronic” instead of “repeated.”

These points have now been corrected.

15. Lines 464-465: Delete the last “s” in both of these phrases: “men prefers” and “women prefers”

This has been corrected.

16. Line 465: I disagree with the assertion that “women prefers more masculine faces.” For instance, even the review article cited in support of this mentions the many nuances (e.g., contextual moderators) that would qualify such a broad assertion as well as findings in the opposite direction. Thus, I think at least an indication that these caveats are acknowledged should be provided. This is important because it will make the authors’ explanation of some of their effects more tentative, which I think would be more balanced at this stage of our knowledge about the topic.

We agree and this was corrected in the sentence lines 460 to 462 page 20.

17. Line 478: I do not understand the usage of “escalated”. How do these factors escalate (i.e., increase in intensity) to anything? I am not a native speaker but this does not make sense to me.

We have replaced “escalated” by “lead to”.

18. Line 536: I think this heading is not grammatical because of the phrase “evolutionary triggered.” The second paragraph under this heading seems unrelated to the heading.

The second paragraph has been deleted and integrated into the Introduction (see response to the following question).

19. Line 547: If the present study aimed to replicate reference #8, why not mention that in the introduction?

This is now clearly indicated in Introduction, lines 82, 98 to 99, 101 to 103 page 4. This is the paragraph deleted in question 18 which has been incorporated here.

20. Line 574: Was this analysis reported anywhere? If no intention to report, then maybe the authors could add a note that the details are available upon request. Better, it could be added to the supplementary materials.

This analysis is now presented in the new Table S6.
